# High-scale random access on DNA storage systems

**DOI:** 10.1093/nargab/lqab126

**Published:** 2022-01-14

**Authors:** Alex El-Shaikh, Marius Welzel, Dominik Heider, Bernhard Seeger

**Affiliations:** Department of Computer Science, University of Marburg, Marburg 35037, Germany; Department of Computer Science, University of Marburg, Marburg 35037, Germany; Department of Computer Science, University of Marburg, Marburg 35037, Germany; Department of Computer Science, University of Marburg, Marburg 35037, Germany

## Abstract

Due to the rapid cost decline of synthesizing and sequencing deoxyribonucleic acid (DNA), high information density, and its durability of up to centuries, utilizing DNA as an information storage medium has received the attention of many scientists. State-of-the-art DNA storage systems exploit the high capacity of DNA and enable random access (predominantly random reads) by primers, which serve as unique identifiers for directly accessing data. However, primers come with a significant limitation regarding the maximum available number per DNA library. The number of different primers within a library is typically very small (e.g. ≈10). We propose a method to overcome this deficiency and present a general-purpose technique for addressing and directly accessing thousands to potentially millions of different data objects within the same DNA pool. Our approach utilizes a fountain code, sophisticated probe design, and microarray technologies. A key component is locality-sensitive hashing, making checks for dissimilarity among such a large number of probes and data objects feasible.

## INTRODUCTION

The amount of digital data created worldwide is growing exponentially and at an ever-increasing pace. Despite the growing storage density, today’s storage technologies such as HDD and tape are out-paced and cannot keep up with the growth of these data rates. It is estimated that by the year 2025 we will have reached 175 Zettabytes globally of total stored data (1). Nearly 80% of it is considered ‘cold’ data that is not frequently accessed, making it an optimal candidate for DNA storage. Furthermore, storing data into DNA has become more prominent due to its unmatched storage density. The theoretical data density for DNA is estimated to be around 455 EB per gram (2), about 50 million times higher than that of traditional media such as HDDs. The durability of DNA plays another important role, exceeding centuries, while HDDs and tapes require replacement every 5 or 30 years, respectively (3). When storing digital data into DNA, one first needs to map digital bits to DNA bases, the building blocks of DNA. There are four bases for DNA: adenine (A), thymine (T), cytosine (C) and guanine (G). All sequences are not equally viable, so this mapping cannot be done in a naive way. For example, sequences with multiple repeats of the same base (*homopolymers*), e.g. ‘AAAAAAA’ are more error-prone in the sequencing (reading DNA) process (4) and thus can not be used. Following the mapping process, the resulting DNA strands can be synthesized (writing DNA). To read data from DNA storage, it is first necessary to sequence the target DNA strands. A significant aspect making DNA storage a promising future alternative to traditional hardware is the sequencing cost, which has been dramatically declining over the past years. But even when considering the sharp decline in costs associated with DNA data storage, without the ability to access specific information directly, DNA data storage would still be too time and cost-intensive to be a viable alternative to traditional long-term storage devices. In this paper, we will address the following challenges in parallel:

1. How do we encode information into DNA at very high densities and minimize errors?
2. How do we enlarge the DNA address space and enable efficient random access at a large scale?

Considering (1), recent studies outline challenges in lossless information retrieval (*perfect recovery*) from DNA. However, synthesizing and sequencing errors have been reduced dramatically, and sequencing costs have dropped by a factor of nearly 100 000 in the past few years. We will show that it is possible to encode information into DNA with little redundancy and no errors. Challenge (2) remains an open question. In particular, most of the current DNA storage systems only provide up to ≈10 different data objects that can be addressed within a single DNA pool. This restriction is due to the biochemical limitations of the polymerase chain reaction (PCR). The main problem with addressing data objects is the limited number of available primers.

Primers are special pre-known sequences used to identify a single DNA strand within a pool uniquely. Usually, each DNA strand has a unique primer pair attached to each of its ends, which unambiguously identifies that strand. These primers are also used to amplify, i.e. copy the sequences for sequencing and synthesizing purposes. There are extensive restrictions considering primers and the target DNA strands that contain the information. For example, primers cannot overlap with any of the DNA strands within the same library.

This disadvantage is often mitigated by splitting the DNA library into multiple DNA pools, each physically separated and thus treated as separate DNA libraries. This enables up to ten addressable data objects per pool, allowing us to address a total of 10*n* objects for *n* pools. However, the usage of multiple pools introduces additional overhead and greatly decreases the information density, which is a crucial advantage of DNA. It was demonstrated that one could use special DNA prefixes to address DNA strands that extend the address space beyond ten (5). The authors designed a system called ‘DORIS’ that offers a maximum of 12 000 addresses. Nonetheless, this address space is insufficient considering the theoretical capacity of DNA storage systems, even when assuming that each DNA strand represents a single data object. Most recent studies (2,3,6–11) do not support random access on their DNA storage medium (12) or it is very limited. To reduce errors, these systems require a 5–3000-fold physical and logical redundancy, which leads to a substantial reduction of storage density. In addition, most of the works mentioned do not encode information such that the resulting DNA is sufficiently stable for long-time archival or perfect decoding. In particular, only a few encoding schemes are aware of, e.g. 50% GC content and minimization of complex secondary structure formation such as hairpins. It was often necessary to increase the sequencing coverage to perfectly decode the data, i.e. multiple reads of the same sequence and additional redundancy were needed for retrieval.

In this paper, we present a proof-of-concept for a method that enables encoding arbitrary digital data into DNA and supports random access to up to millions of addressable data objects within the DNA.

## MATERIALS AND METHODS

### General biochemical restrictions on DNA

DNA can form complex shapes as it winds and coils around itself. The shape of DNA depends on its nucleotides’ arrangement and the surrounding temperature. Each possible shape is referred to as a secondary structure, and a single DNA strand (or double-helix) can have different secondary structures depending on the temperature. Note that sequencing machines fail to read DNA that forms complex secondary structures, and thus these structures have to be minimized. To obtain stable DNA, we have to consider the following biochemical constraints (7,13):

(C1) GC content (number of G’s and C’s) should be around 50%.
(C2) Consecutive repeats of the same nucleotide (*homopolymers*) should be minimized.
(C3) Similarities between primers’ sequences should be minimized.
(C4) Similarities between strands and primers should be minimized.
(C5) Similarities between strands’ sequences should be minimized.
(C6) Secondary structures such as hairpins should be minimized.

Constraint C1 and C2 are known to be highly correlated with sequencing and synthesizing errors. Moreover, G and C form three hydrogen bonds while A and T form two, with each hydrogen bond requiring energy to break. Thus, G and C bonds are more thermostable than A and T bonds. Despite this, the hydrogen bonds themselves do not significantly increase DNA stability, which is primarily achieved by molecular interactions referred to as base stacking (14). A uniform distribution of the number of A’s, T’s, C’s and G’s yields a more stable DNA in general. Constraints C3 and C4 assure that PCR is targeted at selected primers and does not falsely amplify subsequences of other DNA strands. C5 minimizes cross-hybridization: If the DNA pool contains fragments that overlap, similar strands compete in hybridization and partially bind to the wrong halves. This can result in altering the DNA pool and hindering the correct hybridization. Fulfilling C6 ensures that DNA is stable enough for further storing, sequencing and synthesizing.

### Microarrays and probe design

Microarrays, often called DNA chips, are solid surfaces usually made of glass used to identify several hundred to thousands of genes simultaneously. Typical applications are gene-expression analysis, detection of diseases such as cancer, genotyping, and other medical diagnostics (15–17). The solid substrate of a microarray contains a large number of spots/sites, each smaller than 200 microns (18,19) where DNA can be immobilized to. This process is referred to as ‘DNA downloading’, and each DNA sequence that is downloaded is referred to as a *probe* or *barcode*. Probes are usually single-stranded DNA sequences. Note that immobilization does not affect the correct binding ability of the probes, even when one end is fixed to the surface of the array. Furthermore, the DNA chip can comprise up to millions of sites in an area of 1−2 cm^2^. Additionally, some arrays are designed such that already immobilized probes can be replaced. An alternative to microarrays is the bead capture method (20). Microarrays and bead captures are used to select specific sites within a DNA pool and thus can be used interchangeably with marginal differences. Throughout this paper, we will further only use microarrays and not bead captures.

Microarrays are flexible, and the same array can be reused and adapted to new projects without inducing high replacement costs (21). Generally, a microarray answers the question, ‘Is a specific DNA sequence contained in the library?’. To answer this question, we follow the steps below:

1. Identify DNA sequences of interest.
2. Immobilize (download) corresponding probes onto the microarray sites.
3. Place the DNA library onto the microarray.
4. Wash the microarray and insert it into a scanning device.

**Figure 1.**
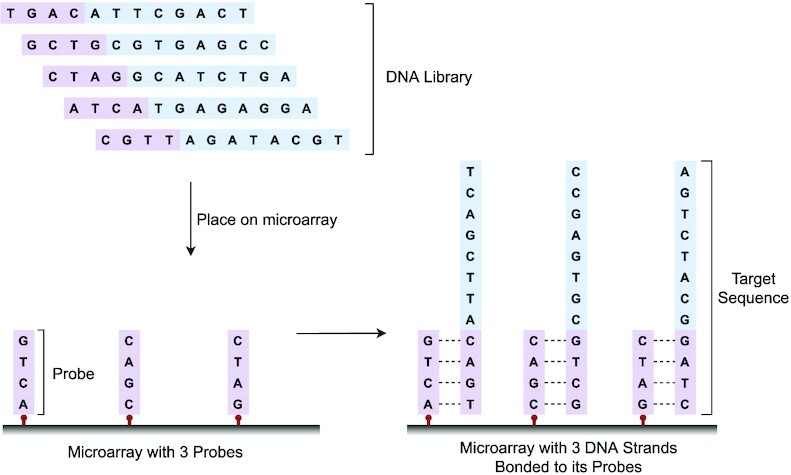
A microarray with three probes selecting the corresponding three targets out of a DNA library of five DNA strands.

In step (1), we need to identify one or more DNA sequences of interest and will refer to these sequences as targets. For example, we would select genes associated with a specific trait, disease, behavior, etc. Then, we define our probes as the complementaries to chosen targets. For example, if our targets were {TGAC, GCTG, CTAG}, then our probes would be {ACTG, CGAC, GATC} respectively. Since probes are single-stranded and targets are usually stored as double-stranded DNA, probes are also contained in the target strands. In step (2), we download the probes to our microarray by synthesizing them onto the microarray’s sites. In step (3), under certain thermal conditions, we pour our DNA library over the microarray, enabling the targets to hybridize to the probes of the microarray. After that, targets that bonded weakly in step (4) due to mismatches are washed away, leaving only the strongly bonded targets. Finally, the remaining bonded targets can be sequenced and retrieved *in-silico*. In Figure 1, five DNA strands are placed onto a microarray that has three probes {ACTG, CGAC, GATC} downloaded to. As shown, the array successfully selects the strands that contain its probes’ complementaries as subsequences and ignores the strands that mismatch with the probes. Target sequences can be very long when short subsequences are sufficient to unambiguously identify the targets. Hence, probes are usually chosen as shorter subsequences of the complementary targets. Note that targets that hybridize to their corresponding probes can be read out entirely, not only the bonded region. Probes are typically around 18−25 bp in length, each theoretically allowing us to address up to 4^25^ targets. Nevertheless, to reduce cross-hybridization noise, probes have to be sufficiently different from each other and thus require careful design. This property is referred to as the *specificity* of probes. The higher the specificity, the less cross-hybridization noise of probes. Additionally, all DNA sequences, including probes, have to fulfill the constraints C1, C2 and C6. Factoring in all constraints, an appropriate probe design results in a noticeable reduction of available sequences. For example, if probes are too similar, target regions complementary to the probes would compete while hybridizing and produce false positives.

Furthermore, the absence or presence of a target can be directly determined from the microarray without sequencing. This is done by labeling the targets with a light-sensitive chemical, such as a fluorophore that re-emits light after excitation. After hybridization, these targets cause light emission, creating an image captured with a specially-designed camera. The obtained image contains the light intensity for each spot of the array, indicating the presence or absence of the respective target. The strength of this light signal is additionally used to calculate relative concentrations of target DNA. In this work, we do not need to mark or label the targets and only consider sequencing the bonded sequences for further *in-silico* analysis. Since a single microarray can contain up to millions of different probes, it allows millions of tests in parallel and is highly scalable. In other words, one can search and find millions of specific sequences in a given DNA pool in one operation simultaneously. In addition, only targeted DNA strands are sequenced, and thus sequencing costs can be further reduced.

### Approximating DNA similarities

Limiting overlaps between Info-DNA and probes are crucial for maintaining low hybridization noise between DNA strands and probes. Therefore, in addition to the biochemical constraints we mentioned before, we will add the following constraint:

(C7) Similarities between Info-DNA and probes should be minimized.

A naive approach to identifying similar sequences is not scalable as it requires every sequence to be tested against all the other sequences. To calculate the similarity of two DNA sequences efficiently, we implemented locality-sensitive hashing (LSH) that enables approximating the similarity with low computational overhead (22). In particular, we approximated the Jaccard similarity for DNA sequences utilizing MinHash (23–26). To calculate the Jaccard similarity of two DNA sequences, we first convert each of the sequences to a set of *k*-mers. Let *S_k_*(*q*) be the *k*-mer set of sequence *q*, i.e., the set that contains all continuous subsequences of *q* of length *k*. For example, the sequence *q* =ACTACC, is mapped to the 3-mer set *S*_3_(*q*) ={ACT, CTA, TAC, ACC} and for *k* = 4, the same sequence is mapped to $S_{4}(q)=\lbrace \tt {ACTA}, \tt {CTAC}, \tt {TACC}\rbrace$. After that, we calculate the Jaccard similarity (*sim_k_*) of two sequences *q*_1_ and *q*_2_ as follows:

(1) \begin{eqnarray*} sim_{k}(q_1, q_2) = \frac{|S_k(q_1) \,\,\cap \,\, S_k(q_2)|}{|S_k(q_1) \,\,\cup \,\, S_k(q_2)|} \end{eqnarray*}

Furthermore, for two sequences *q*_1_, *q*_2_, a threshold *t*, and an approximation factor *c* > 1, LSH uses *r* hash functions *h*_1_, ..., *h_r_* from a function family $\mathcal {F}$, for which the following holds:

(2) \begin{eqnarray*} d_k(q_1, q_2) \,\,\le \,\, t \,\,\,\,\Rightarrow Pr[h(q_1) = h(q_2)] \,\,\ge \,\, p_1 \end{eqnarray*}

(3) \begin{eqnarray*} d_k(q_1, q_2) \,\,\ge \,\, c \cdot t \,\,\,\,\Rightarrow Pr[h(q_1) = h(q_2)] \,\,\le \,\, p_2 \end{eqnarray*}

where *Pr* denotes the probability, *d_k_* = 1 − *sim_k_* is the *distance* function, and $h\in \mathcal {F}$ is a randomly selected (uniformly) hash function. If the distance of *q*_1_ and *q*_2_ is below threshold *t*, the probability of *q*_1_ and *q*_2_ mapping to the same hash value is at least *p*_1_. Conversely, if the distance of *q*_1_ and *q*_2_ is above *c* · *t*, the probability of *q*_1_ and *q*_2_ mapping to the same hash value is at most *p*_2_. Moreover, we amplify our LSH by the *OR-construction* that reduces false negatives (27,28). Every *m* hash functions further refer to a *band*. Hash values of a band of a single sequence are combined to a *signature*, which is mapped into a band hash table. Therefore, LSH finds sequences of which signatures match in at least one band. The resulting LSH algorithm approximates Equation (1) and is parameterized by the number of hash functions *r*, the *k*-mer length *k*, and the number of bands $b=\frac{r}{m}$. As demonstrated in Supplementary Figure S6, when the number of bands is small, i.e., the signatures are composed of many hash values (large *m*), the signatures of two sequences are less likely to match in any band, even if the actual similarity is high. However, if *b* is large (small *m*), the signatures only contain a few hash values and thus are more likely to match in at least one band, even if the actual similarity is low.

By utilizing LSH for similarity checks, we can quickly decide if a new DNA sequence is similar to a given collection of DNA sequences or not. Furthermore, we use a single primer pair for our DNA sequences, which would allow the amplification of the whole library if necessary. Note that the primer sequences also have to be avoided while encoding the data. We will first explain how we generated probes, then briefly introduce fountain codes, which is the basis of our encoding scheme. After that, we will describe the encoding pipeline that produces DNA that fulfills all constraints C1 to C7 and enables random access.

### Probe generation

To provide the keys for our encoding approach, we need to generate a probe for each value. Since the probes are computed prior to encoding the data, we do not need to check for their similarities to Info-DNA sequences at this stage. As presented in Algorithm 1, we specify a GC content range [gcMin, gcMax] of which the probe *p* will be sampled.


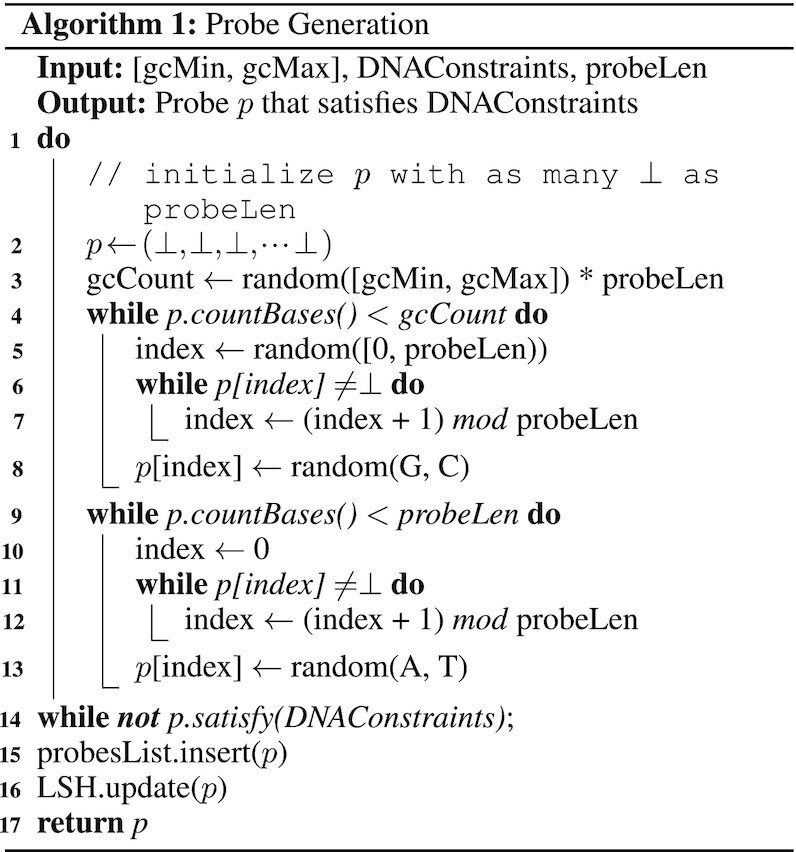


Next, an empty sequence of length probeLen is initialized in line 2. In line 3, we calculate the number of G’s and C’s the probe will have. Then, from line 4 to line 8, we fill exactly gcCount indices of *p* with randomly G or C. After that, we fill the rest of *p* with randomly A or T in line 9 to line 13. This allows a uniform distribution of the different bases and yields more stable DNA. Finally, if *p* does not satisfy DNAConstraints, which resemble constraints C1, C2, C4 and C6, we start over until *p* eventually fulfills the given constraints. Note that constraint C4 can be efficiently checked by utilizing LSH. If the generated sequence satisfies these constraints, we insert it into the list of probes (probesList) and update LSH. This process is repeated until a desired number of probes is generated.

### Fountain code overview

**Figure 2.**
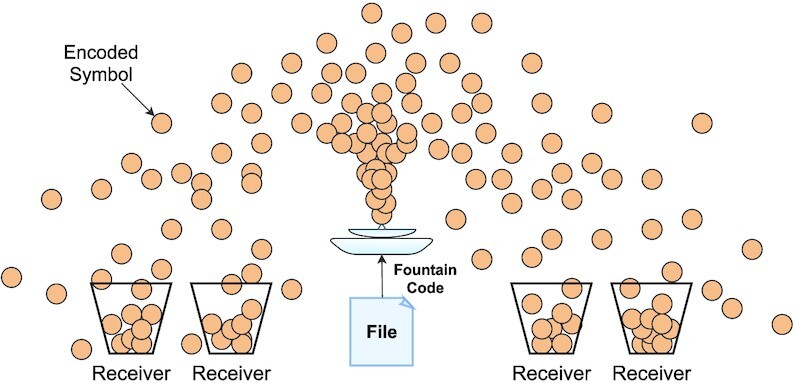
A fountain code generating encoded symboles captured by four receivers via a lossy channel.

Fountain codes are a class of rateless erasure codes that generate a potentially infinite sequence of encoded symbols for a given *k*-symbol message. In particular, they can create an arbitrary amount of redundancy symbols that can be used to recover the source message. For a *k*-symbol source message, any subset of length *k* + ϵ encoded symbols can be used to fully recover all *k* source symbols with high probability, where ϵ is called the ‘overhead’ and is usually a small number. The idea is to enable senders to send encoded symbols (*packets*) over a network, where the receiver can signal its sender once the message was successfully decoded. In Figure 2, a fountain code is deployed that sends packets over a lossy channel to four receivers. Note that in order to recover the original message, each receiver requires *k* + ϵ arbitrary packets. Moreover, faulty packets that contain unrecoverable errors can be ignored by the receiver. Even if some of the packets were lost during transmission, the sender could continue sending packets (overhead) until the signal from the receiver is captured.

Luby Transform (*LT*) codes were the first fountain codes, published by Michael Luby in 2002 (29) that are near-optimal erasure codes. LT codes were further improved (30), and the latest improvement of this class of codes is the RaptorQ (*RQ*) code (31) and was used to encode and decode our data. The key advantages of LT codes are the simple encoding and decoding algorithms, which are based on the exclusive-or operation and have linear time complexity. There is a trade-off between overhead (redundancy) and recovery probability as follows:

- Overhead $\varepsilon = 0 \Rightarrow Pr[^{\prime \prime }recovery^{\prime \prime }] >99\%$
- Overhead $\varepsilon = 1 \Rightarrow Pr[^{\prime \prime }recovery^{\prime \prime }] >99.99\%$
- Overhead $\varepsilon = 2 \Rightarrow Pr[^{\prime \prime }recovery^{\prime \prime }] >99.9999\%$

A typical use case for RQ codes is data transmission over lossy networks. If the data were successfully transmitted, the receiver is very likely to decode and fully recover the message. Even if some part of the message was lost during transmission, the receiver could still recover the message if sufficient overhead was provided.

### RQ probe-aware encoding pipeline (RQPAP)

The original RaptorQ code takes a binary data object as input and outputs a binary stream of packets, which can be decoded to recover the input object. Typically, for a source message with source symbols (*x*_1_, …, *x_k_*), the encoder generates a stream of encoded symbols (*y*_1_, *y*_2_, …) such that *x_i_* = *y_i_*,  *i* ≤ *k*. The first *k* encoded symbols are equal to the source message, and the following symbols *y*_*k* + *i*_,  *i* > 0 are repair symbols (*overhead*). Hence, only if transmission of the original message was faulty, i.e., the receiver did not signal to the sender, we will need repair symbols to recover errors. This variant of fountain codes is referred to as *systematic* codes. To further encode the output stream to DNA, we map every two consecutive bits to a nucleotide (base). For example, we map (01 10 10 00 11) to (TCCAG) and (00 00 00 00 00) to (AAAAA). Note that if the original message contains a vast number of consecutive 0’s or 1’s, the resulting DNA message will inevitably contain long homopolymers, which are highly error-prone. To make our RQPAP work for arbitrary binary data objects, even for ones containing long chains of 0’s or 1’s, we used a non-systematic RaptorQ code, where the first *k* symbols of the encoder are ignored, and only repair symbols are considered. This is key since repair symbols are computed from a range of source symbols (see Supplementary Figure S7) and are thus less susceptible to being encoded as unwanted homopolymers. In the next section, we will show how we generate DNA packets for a given data object. We will then show how to combine DNA packets of a single data object to a DNA sequence (Info-DNA). As mentioned above, we can use a single primer pair for all generated DNA strands, which will be used to amplify the pool as a whole, and is not used to perform random access. Note that according to the constraints C1 to C7, overlaps of two different Info-DNAs, overlaps with probes, and with the primer pair should be minimized. We will call these constraints *encoding constraints*. Hence, we utilized the RaptorQ code for encoding a given data object while effectively solving the present encoding constraints. That means it iterates over its unbounded stream of DNA packets (*y*_1_, …) and selects a subsequence of that stream (*y_i_*, ..., *y_j_*),  *j* > *i*, that is decodable and satisfies all of the encoding constraints. For example, in Supplementary Figure S8, (*y*_2_, *y*_3_, *y*_4_) is a decodable subsequence that satisfies all encoding constraints, while (*y*_1_, *y*_2_, *y*_3_) is not decodable but satisfies the encoding constraints, and (*y*_1_, *y*_2_, *y*_3_, *y*_4_) is decodable but does not satisfy the encoding constraints.

### Generating DNA packets

To generate DNA packets for a given data object, we split the object into equally sized chunks, as depicted in Supplementary Figure S7. Then, we randomly select a pre-defined number of chunks, which are fed into the RQ’s encoder that creates a binary packet. Note that these are repair packets and do not contain the plain source message. Next, every two consecutive bits are mapped to a corresponding nucleotide by the mapping rules (00↦ A, 01↦ T, 10↦ C, 11↦ G), resulting in a DNA packet. The obtained DNA packet is then parsed, and DNA constraints C1, C2, C3, C4, C5 and C7 are checked. Suppose the DNA, e.g. contains too long homopolymers, too low or high GC content, or a significant overlap with primers, probes, or other Info-DNA. In that case, the packet is discarded, and the next packet is computed. RQ can generate a potentially infinite number of packets, of which the first *k* + ϵ packets that allow decoding and satisfy the constraints are selected. Note that computing the overlaps is done efficiently by calculating similarities using LSH and can be scaled up without noticeable computational overhead. The advantage of RaptorQ is that it can generate a large variety of binary packets in parallel and thus eventually produces a DNA packet that fulfills all encoding constraints. Constraint C6 is not considered at this step since multiple packets are further combined to create an Info-DNA strand.

### Combining DNA packets to DNA strands

To create the Info-DNA strand for a given data object, we must combine multiple DNA packets generated as described above. The resulting Info-DNA strand needs to contain enough DNA packets to recover the input data object fully. At the final stage, a probe is annealed to its beginning. As illustrated in Algorithm 2, we first initialize the output strand *s* with an empty sequence in line 1. Next, a DNA packet is generated and appended to *s*. While *s* does not contain enough packets to fully recover the input data object, the algorithm loops, appending an additional DNA packet every time. Eventually, *s* contains a sufficient number of packets, allowing for successful decoding of the object. Note that canDecode can be configured such that it only returns positive once *s* is decodable and contains a certain overhead. The given constraints (DNAConstraints) are then evaluated in line 6. If *s* fulfills them, probe *p* is annealed to its beginning. At this point, the strand resembles the fully encoded data object. Furthermore, the mapping table (DOI ↦ probe) and LSH are updated in line 8 and line 9. Note that we check the GC content (C1) for each DNA packet, which yields a more uniform distribution over the resulting Info-DNA. Parameters such as similarity thresholds, GC content, DNA stability (max homopolymer length, secondary structure stability, etc.) can be tweaked and adapted. Moreover, we provide a tunable redundancy parameter for the creation of encoded data objects. Nevertheless, if some of the parameters, e.g. similarity threshold, are too strict, the pipeline could loop forever, failing to encode the given objects successfully. Additionally, if the similarity threshold is set very low, most sequences will get checked by LSH, thus slowing down the encoding speed. Furthermore, each data object can be encoded in parallel, which significantly increases the speed. This requires the probes’ list, mapping table, and LSH to be synchronized, inducing a minimal overhead.


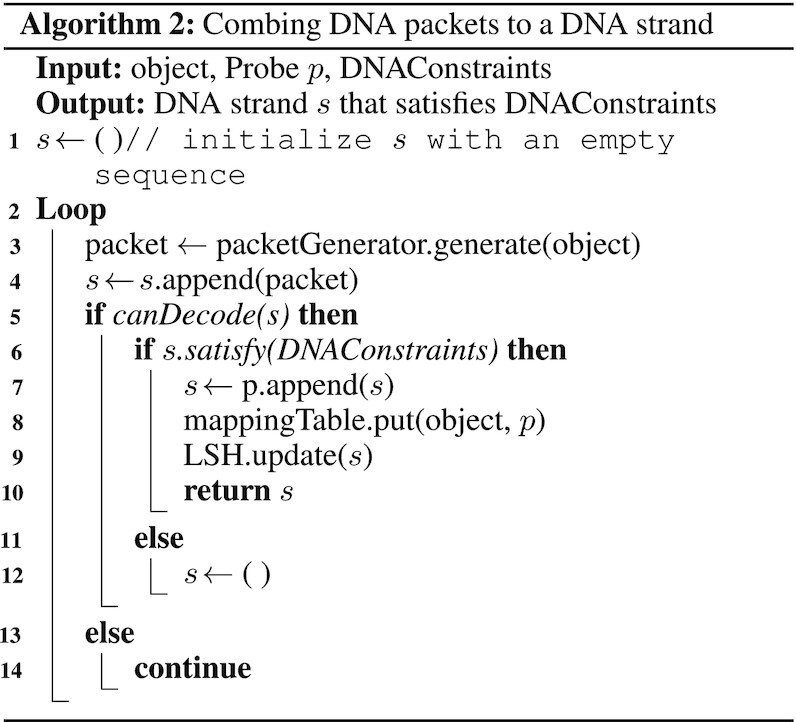


## RESULTS

### DNA-based storage system

**Figure 3.**
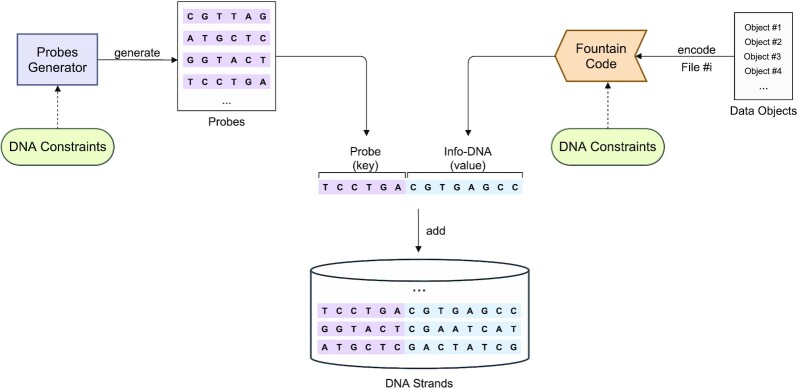
DNA strands are computed by annealing a probe (key) to an encoded data object (value). The probe is obtained from the list of probes generated by the probes generator and the corresponding value (Info-DNA) is computed by the fountain code.

We organized our DNA pool as follows: each DNA strand is composed of a probe and an information-carrying part (*Info-DNA*). The probe acts like a primer and uniquely identifies the strand but is not used for PCR amplification. For information encoding, we use a fountain code, i.e. RaptorQ code (*RQ*) that enables variable forward error correction (FEC) and redundancy. Finally, a microarray with printed probes is used to select and then sequence a target DNA strand. To efficiently calculate unwanted overlaps of DNA sequences, we implemented a locality-sensitive hashing (*LSH*) algorithm incorporated into the probe design and the fountain code’s pipeline. We used LSH to approximate the Jaccard distance of two DNA sequences as described in Materials and Methods. Furthermore, our encoding pipeline incorporates probes that were computed before encoding the data. Each data object is mapped to a single probe, and the *mapping table* (data object ↦ probe) is stored *in-silico*. Each data object could be referred to by a unique data object identifier (DOI), i.e. a number that unambiguously identifies that data object. Hence, the *mapping table* would store a collection of (DOI ↦ probe) pairs. Moreover, the encoding scheme must be aware of the probes’ sequences and avoid overlaps, i.e., similarities between DNA sequences of encoded data (*Info-DNA*) and the probes. The resulting DNA strands, which resemble the final DNA sequences, are each composed of a probe (*key*) annealed to an Info-DNA sequence (*value*) as shown in Figure 3. This kind of organization is therefore called *key-value* store.

### Random access

**Figure 4.**
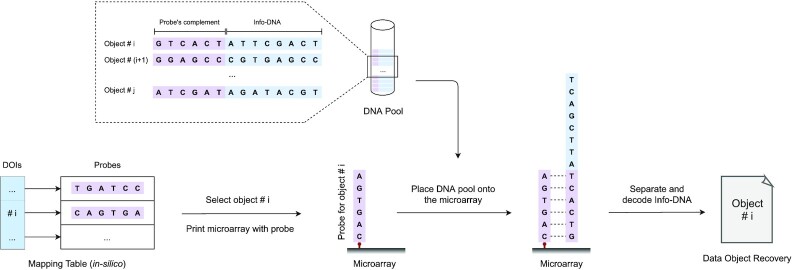
Performing random access on a data object using a microarray.

As described above, the resulting DNA strands resemble a collection of key-value pairs, which can get synthesized and stored in a DNA library or even in a single pool. We refer to random access as a random read operation. In other words, random access resembles a search operation that opts to find and read a specific data object (Info-DNA) by its key (probe) within a library of key-value pairs. Since the DNA strands are double-stranded, they contain the corresponding probe and its complement. As illustrated in Figure 4, we need to obtain the specific probe’s sequence that was annealed to the corresponding object. This is determined by performing a lookup in the mapping table *in-silico*. Next, the probe is downloaded (or *printed*) onto a microarray. Then, the whole library (or pool) is placed onto the array to allow the desired target DNA strand to hybridize to the selected probe. Finally, we can sequence the hybridized strand with a scanning device, parse and decode the Info-DNA by RQ code to recover the data. Note that even if parts of the DNA were damaged, RQ can still perfectly restore the data object if sufficient redundancy was implemented.

Furthermore, we can select and sequence multiple data objects in parallel. Since a microarray allows downloading up to millions of probes, we can randomly access up to millions of different data objects simultaneously. Additionally, one can create and manage several microarrays with selected probes that are used frequently prior to random access. Hence, a lookup query can be mapped to an existing microarray and does not require preparing a new array. Some microarrays allow probe replacement and thus enable further adaptation if needed.

### Experimental setup

In order to evaluate the feasibility of our probe-aware encoding approach that provides highly scalable random access on DNA, we ran several *in-silico* experiments. We implemented our probe generation algorithm in Java, and the RQ probe-aware encoding pipeline (RQPAP) was written in Rust. Furthermore, we performed a warm-up run for each experiment before the actual experiment. All our experiments were performed on a server machine equipped with 1TB of RAM and 256 logical processor cores, each operating at 1.5–2.25 GHZ. Furthermore, we first experimentally prove that our probes generator can produce up to millions of probes of high specificity. After that, we evaluate our RQPAP and show that it can encode up to millions of different data objects in a reasonable time.

### Evaluating probe generation

We computed one million different probes that satisfy all DNA constraints we outlined in Materials and Methods. Furthermore, we only allowed the GC content to vary between 40% and 60%, with most of the sequences being close to 50%, and further restricted the maximum length of homopolymers to 5 bp. LSH with parameters (*r* = 200, *b* = 20) was used to determine the similarity of newly encoded probes and data objects to existing ones. Throughout this section, we fixed the length of *k*-mers to *k* = 4, where a *k*-mer is a contiguous subsequence in the DNA of length *k*. These *k*-mers serve for computing the Jaccard distance among DNA subsequences. The parameters *r* and *b* were unchanged for all experiments. Similar sequences, matched by LSH, were checked explicitly via the Jaccard distance, and sequences with similarities that exceeded 60% were discarded. Finally, each sequence was checked for complex secondary structure formation, and only stable (no complex secondary structures) sequences were accepted.

Given a certain number of previously computed probes, we show the computational cost (in milliseconds) for each additional probe computed in Supplementary Figure S1. Longer probes required more time to compute than shorter sequences. This is mainly due to extra computation overhead from the secondary structures formation prediction, requiring more computation for longer sequences. In general, we observed that shorter sequences are less prone to building complex secondary structures.

**Figure 5.**
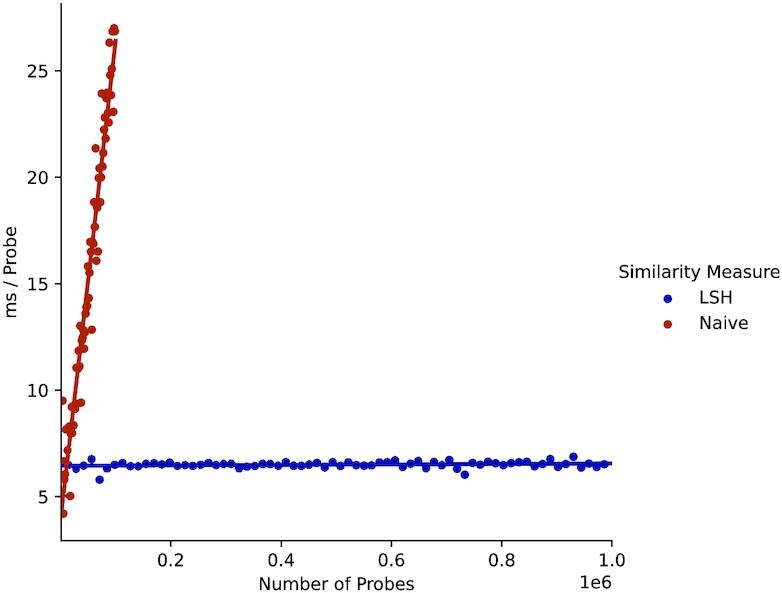
Computation cost per additional probe (LSH versus naive).

In Figure 5, we show how using LSH for similarity checks speeds up probe generation significantly. The probes’ length is set to 60 bp. The blue line represents the computational cost per probe using LSH and the red line (*naive*) for computing the Jaccard distance for each newly created sequence with the previous ones. Note that we made use of the great parallelism available on the testing machine for the naive method, parallelizing similarity computations whenever possible. Nevertheless, despite the high parallelism, we had to limit naive probe generation to a total of 100k due to its large computational overhead. Using LSH for similarity checks resulted in a larger memory footprint, but it allowed us to scale the generation up to several million probes.

### Evaluating probe-aware encoding pipeline

For our experiments on the encoding-pipeline, we used the data sets *ds_1_* and *ds_2_* described in the Supplementary Material. Note that throughout this subsection, we change the *k*-mers length to *k* = 5 while keeping the setting of the LSH parameters unchanged (*r* = 200, *b* = 20). We restricted the maximum homopolymer length to 5bps if not otherwise specified. We required all DNA sequences to have a minimum Jaccard distance of 30% to each other, and similarity checks were only performed on the final DNA strands. First, we computed 100k probes that serve as keys, which were inserted into the LSH table. We used two different LSH instances, one solely for the probes and another for the generated DNA strands of the values. Next, using *ds*_1_, we fed each line into our RQPAP and varied the RQ’s overhead ϵ = 0, 1, 2. As shown in Supplementary Figure S2, a larger ϵ led to significantly more run time. This is due to the great increase in computation time of finding complex secondary structures, as we show in Supplementary Figure S3. The resulting DNA strands for ϵ = 0, 1, 2 contain an average of 133, 161 and 189 bp, respectively. This means, with each increase of ϵ by 1, we gain around ≈20% redundancy. Note that these time measures also include decoding time since we only stop generating DNA packets once the DNA strands were decodable with respect to the specified ϵ.

Furthermore, we wanted to evaluate how varying stringency of the constraints on the DNA strands would affect the overall performance. In Supplementary Figure S4, we encoded the same 100k lines again, setting ϵ = 0 and varying the constraints’ stringency. We restricted the maximum homopolymer length to *Max HP Length* =4, 6, 8, 10. We only observed a slight change in performance. We repeated this experiment several times, which returned the same results (even with different ϵ). Contrary to our expectations, the RQ code could generate a large number of packets without significantly sacrificing performance, of which a sufficient number of the packets were viable according to *Max HP Length*. Moreover, the run time of secondary structure checks was slightly higher for larger *Max HP Length*. In Supplementary Figure S5, we encoded *ds*_1_ in three modes: LSH, mixed and naive. These modes are defined as:

- *LSH*: All similarity checks were performed with LSH.
- *Mixed*: Similarity checks between DNA strands and probes were performed with LSH. However, similarity checks between a newly created DNA strand and previously generated DNA strands are performed naively, i.e. the Jaccard distance was evaluated on each of them.
- *Naive*: All similarity checks were performed explicitly by evaluating the Jaccard distance.

Similar to the results in Figure 5, LSH speeds up encoding time substantially compared to the mixed and naive runs. As expected, the naive run was the slowest, followed by the mixed run, since both require additional evaluations of the Jaccard distance compared to LSH.

**Figure 6.**
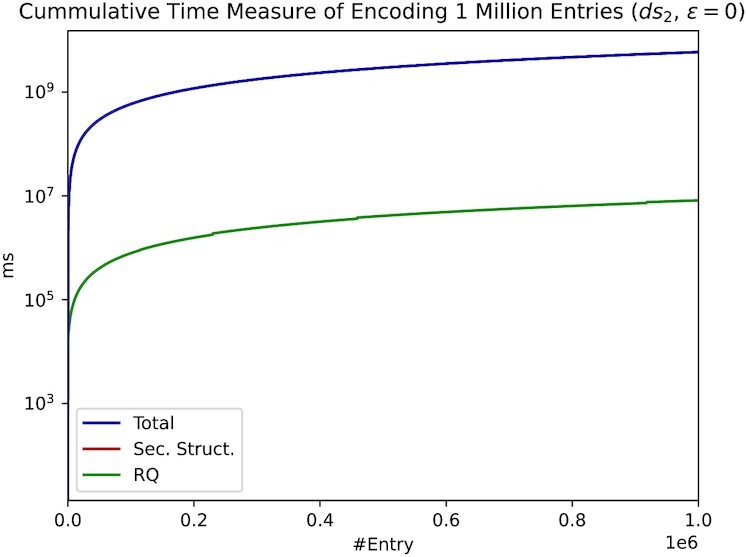
Cumulative time measure for encoding (+decoding) 1 million compressed data entries (lines). The red line is obscured by the blue line because Sec. Struct. time dominates.

In Figure 6, we used 1 million pre-computed probes as keys and encoded *ds*_2_, containing 1 million compressed lines (entries). We show that our approach works on larger data sets, even with 1 million entries. Furthermore, we required a single probe to resemble a key for exactly one data entry or line. This restriction can be further loosened to provide a single key for multiple values. We repeated this experiment with 100k instead of 1 million probes, where a single probe addresses ten data entries. As expected, the results were very similar, and the total run time was lower.

## DISCUSSION

Utilizing DNA as a storage medium offers a great potential to store an enormous amount of data in just a few grams. However, current DNA storage systems fail to provide a sufficiently large address space, and thus the number of directly accessible data objects within a single DNA pool is very limited. In addition, a major challenge for all DNA-based systems is error-free storage and retrieval of the data. While mutation is crucial for living organisms as they evolve and adapt to changing environments, this leads to an unwanted altering of stored information and even unrecoverable losses. To combat this issue, DNA systems typically increase data redundancy or implement error correction codes to ensure data integrity.

This paper proposed a new approach to random access on DNA that supports address spaces up to several million addresses. Instead of relying on primer sequences for random access, we used sufficiently distinguishable and stable probes, for which fewer biochemical restrictions apply. These probes are stored each as a prefix to the associated information on DNA. Since the naive method of checking similarities of DNA sequences is not applicable for generating large address spaces due to its unacceptably high run time, we proposed a scalable method based on LSH and the Jaccard similarity. Our experiments confirm the feasibility of generating millions of probes that obey all conditions required for storing them in a DNA pool. To read information from DNA, we proposed using microarrays, which are capable of selecting specific target DNA strands from millions of other strands. We utilized a fountain code for data encoding that provides forward error correction and mitigates producing error-prone DNA. Our implementation offers various parameters such as redundancy, maximum homopolymer length, and GC content that can be tuned and adjusted. As a proof-of-concept, we showed that our approach is functional and enables massive up-scaling of addresses within DNA. In addition, we submitted our DNA to Twist Bioscience (https://www.twistbioscience.com/) that confirmed the production readiness of our DNA.

In our future work, we will address a notable disadvantage of our approach that still requires a mapping table (DOI ↦ probe) to be stored *in-silico*. We are particularly interested in designing semantic probes that would allow looking up information in an ad-hoc manner. Furthermore, while we presented a promising *in-silico* analysis to achieve a substantial up-scaling of key-value stores on DNA, experiments on real DNA will ultimately prove the true feasibility of our approach. Finally, the examined data sets were in the order of megabytes, but we are interested in applying our method to larger data sets. We look forward to exploring and optimizing our approach in the future.

## DATA AVAILABILITY

The original data sets used are available (download links) in the Supplementary. The preparation (transformation) of the data sets to *ds*_1_ and *ds*_2_ is explained in the Supplementary. Additionally, we provide one million probes that can be directly downloaded from https://github.com/alexelshaikh/Probes.

The RQPAP is available at https://github.com/alexelshaikh/RQPAP.

The probes generator is available at https://github.com/alexelshaikh/PG.

## SUPPLEMENTARY DATA

Supplementary data are available at NARGAB online.

## Supplementary Material

lqab126_Supplemental_File
