# Supplementary material for: High-scale random access on DNA storage systems
Source: NAR Genom Bioinform. 2022 Jan 14;4(1):lqab126. doi: 10.1093/nargab/lqab126 (PMC8829907; doi:10.1093/nargab/lqab126)
Supplement: lqab126_Supplemental_File [file lqab126_supplemental_file.pdf]

Supplementary material for

High-Scale Random Access on DNA Storage Systems

9th of October 2021

Datasets:

1. Dataset$_1$ from [1]:

   Each line contains a date, time, and 13 different values representing air quality measurements within an Italian city.

#	Date	Time	CO(GT)	...
1	10/03/2004	18:00:00	2.6	...
2	10/03/2004	19:00:00	2	...
...	...	...	...	...

Transform

#	Entry
1.1	10032004_Time1=18:00:00
1.2	10032004_CO(GT)1=2.6
...	...
2.1	10032004_Time2=19:00:00
...	...

Transforming Dataset$_1$ from [1] to ds_1 for RQPAP.

As depicted above, we transformed the data set into a new one called ***ds_1***. Only the column named "Entry" was used (line-wise) for the encoding to DNA. We additionally constrained the contents of *ds_1* to the first 100k lines of the transformation. The resulting data set was ≈2.5MB in size, of which each line ranged between 16 and 31 bytes. Note that many of the generated lines held very similar content prior to the encoding. The original data set is available at: https://www.kaggle.com/anveshparashar/airqualityuci?select=AirQualityUCI.csv

2. Dataset$_2$ from the OpenSky Network (https://opensky-network.org/): This data set contains tracking information of aircrafts around the world. Each line contains a specific time, aircraft identification number (icao24), and 14 other values such as aircraft's position (latitude *lat* and longitude *lon*), velocity, etc. Since this data set is very big, we cropped it by only considering the first 1 million lines (without the header), each containing values of the following attributes: time, icao24, lat, and lon. Furthermore, we compressed each cropped line by the same Huffman code. This Huffman code was generated from the cropped data set, of

which the occurrences (frequency) of each byte were calculated. The resulting data set called ***ds_2*** contained ≈20.9MB of data, each line (*entry*) ranging between 16 and 24 bytes. The cropped data set before compression was ≈42.3MB in size. The original data set named "A321_valid.csv" (compressed "A321_valid.csv.xz") is available at: https://opensky-network.org/datasets/publication-data/climbing-aircraft-dataset/trajs/

3. A collection of one million probes is available at: https://github.com/alexelshaikh/Probes

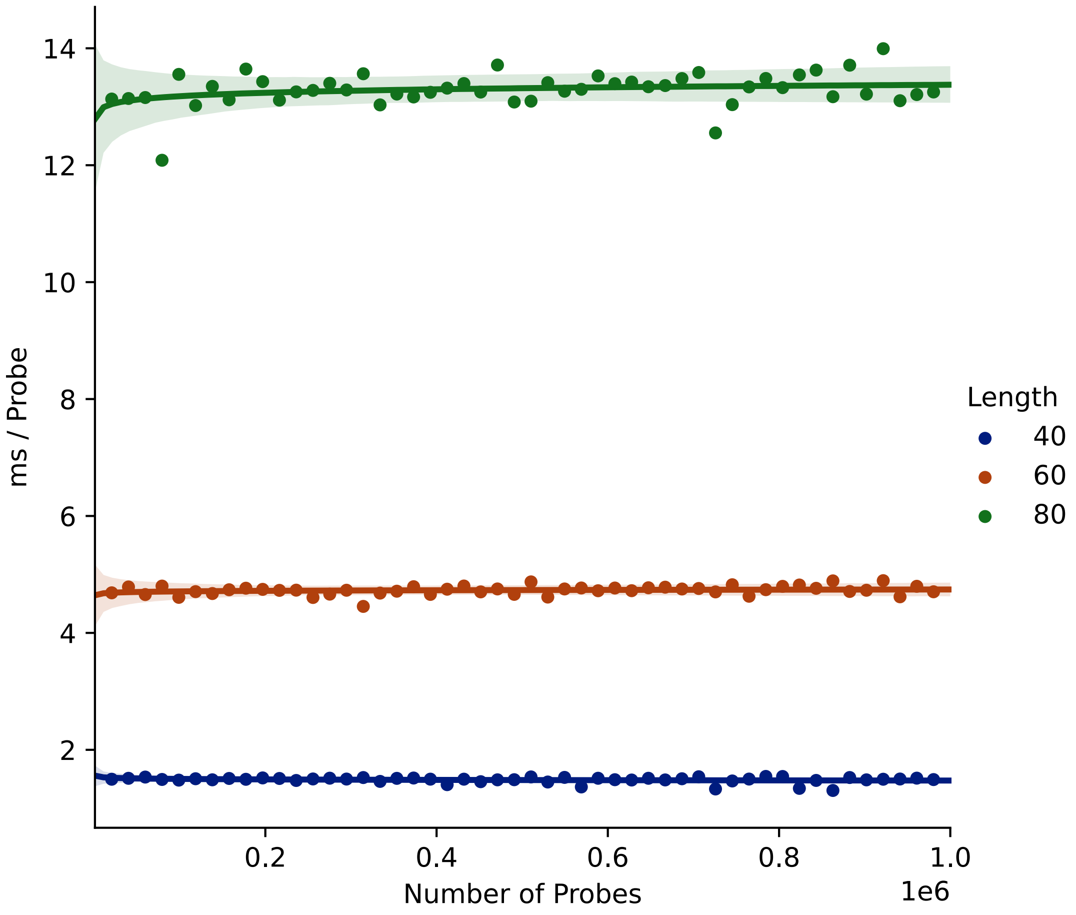

Supplementary Figure 1: *Computation cost per additional probe using LSH.*

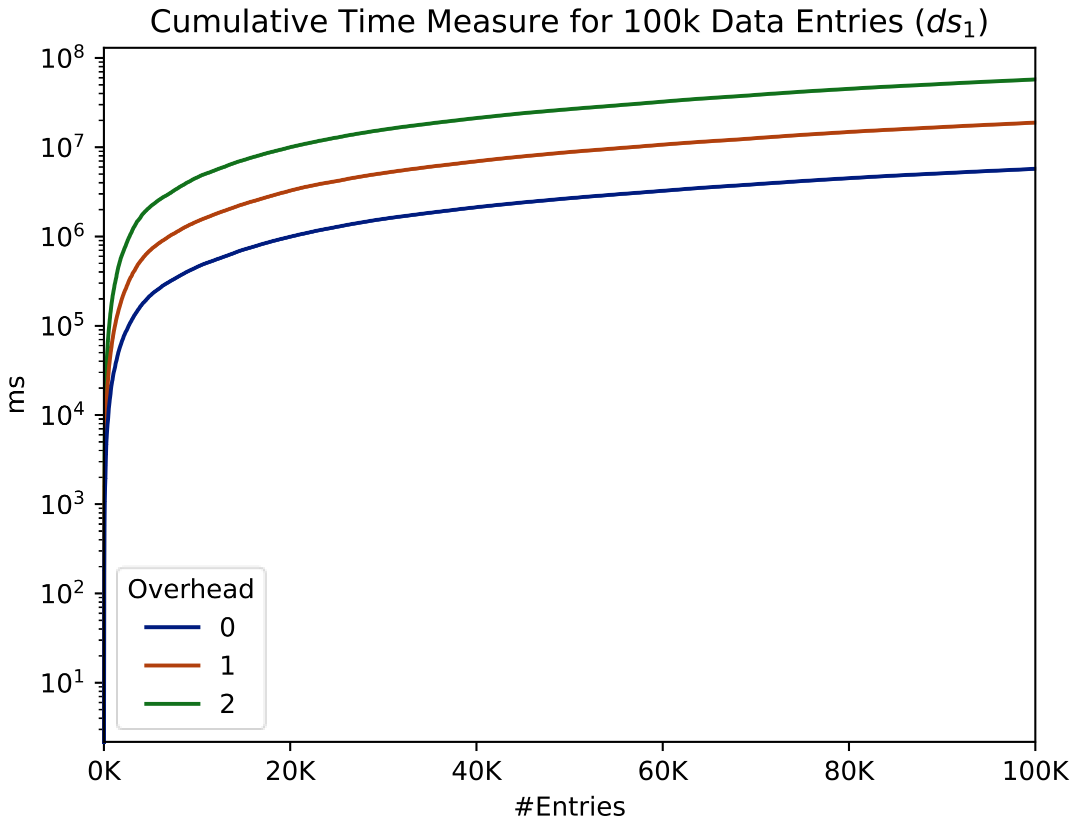

Supplementary Figure 2: *Cumulative time measure for encoding (+decoding) 100k data entries (lines) to DNA strands.*

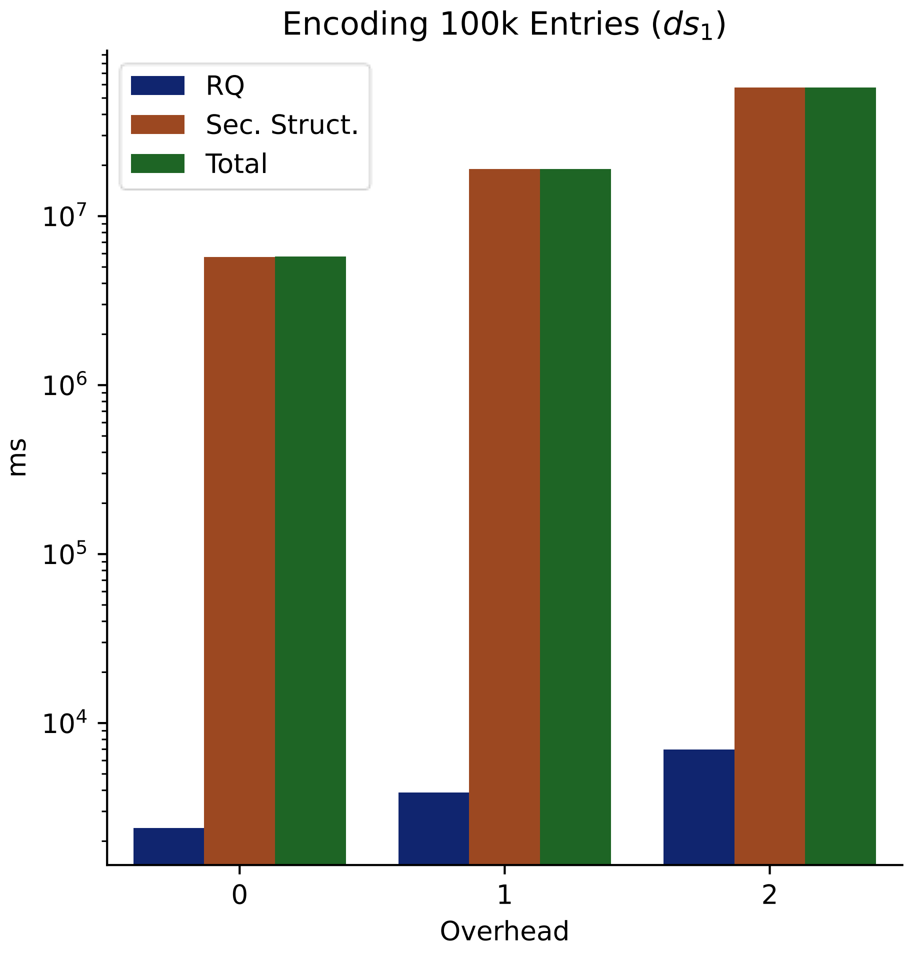

Supplementary Figure 3: The overall times of encoding (+decoding) the 100k data entries (lines) are presented, split in encoding time by RQ Code (RQ), secondary structure checks (Sec. Struct.), and the total time (Total) needed. The secondary structure checks share most of the overall computation time. Notably, the RQ code's time increase is almost negligible. This is due to the high time complexity of secondary structure checks that grow much faster than RQ with increasing DNA strand length (i.e., overhead).

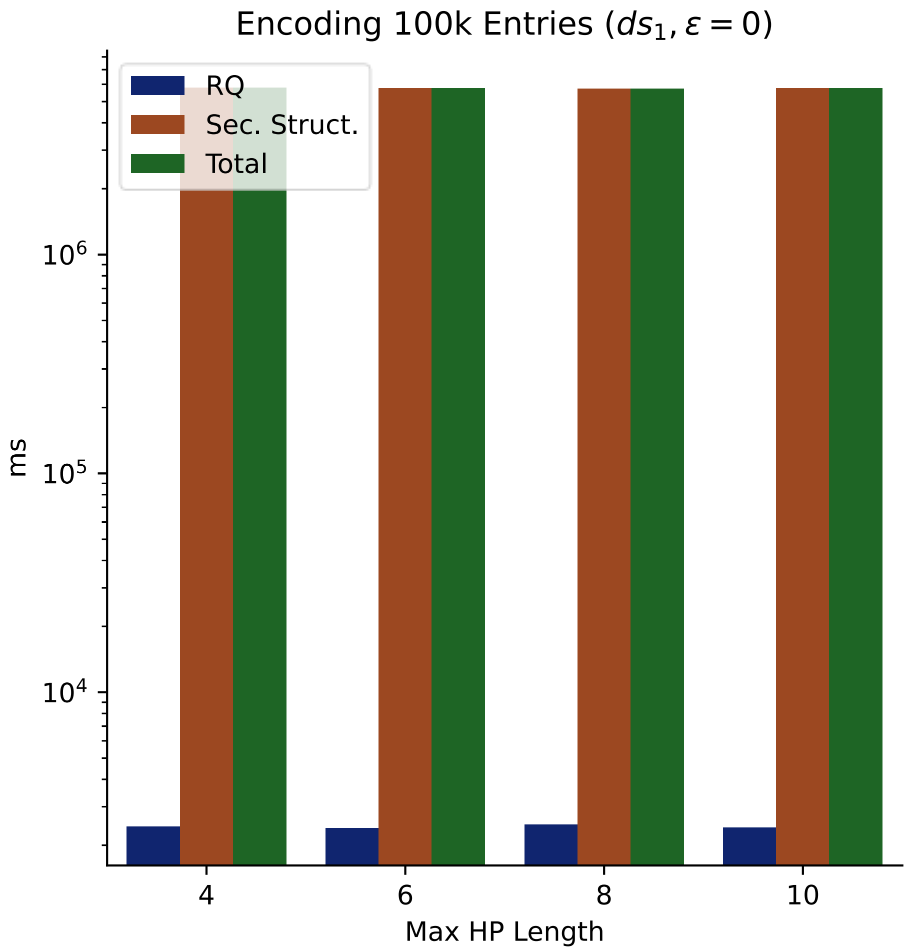

Supplementary Figure 4: *The overall times for encoding (+decoding) 100k data entries (lines) to DNA strands by varying stringency (Max HP Length).*

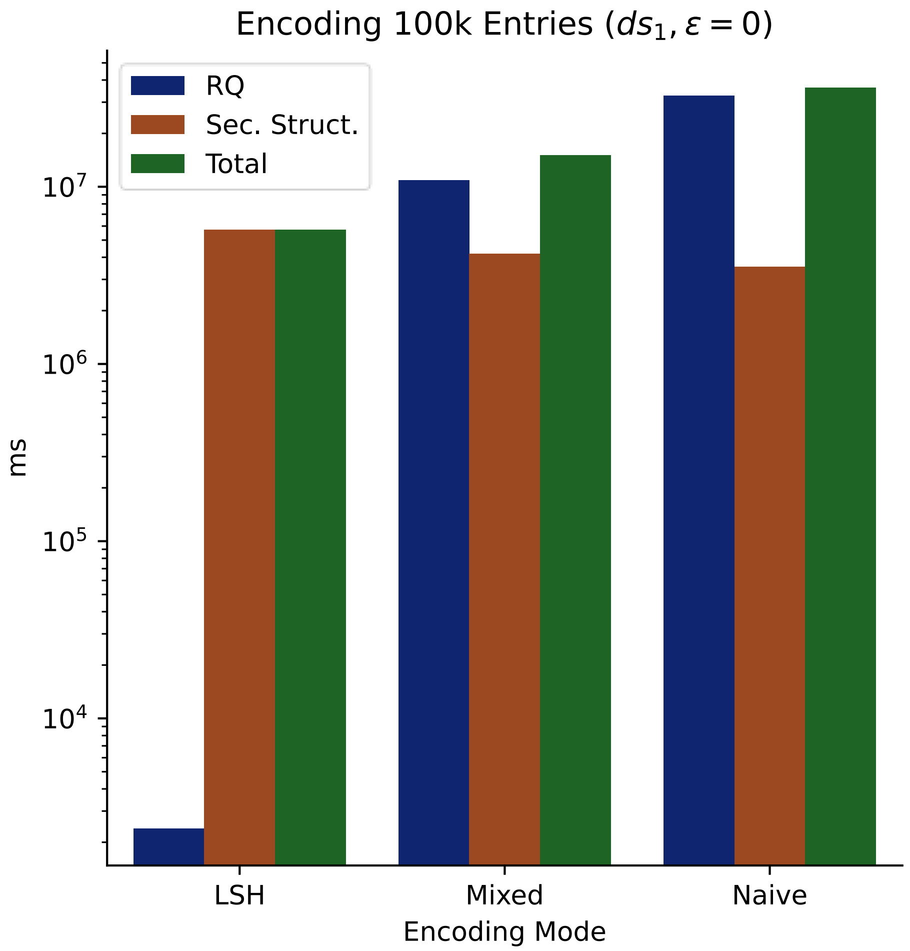

Supplementary Figure 5: *The overall times for encoding (+decoding) 100k data entries (lines) in three different modes. The RQ time increases dramatically (even surpasses Sec. Struct. Time) in "Mixed" or "Naive" mode.*

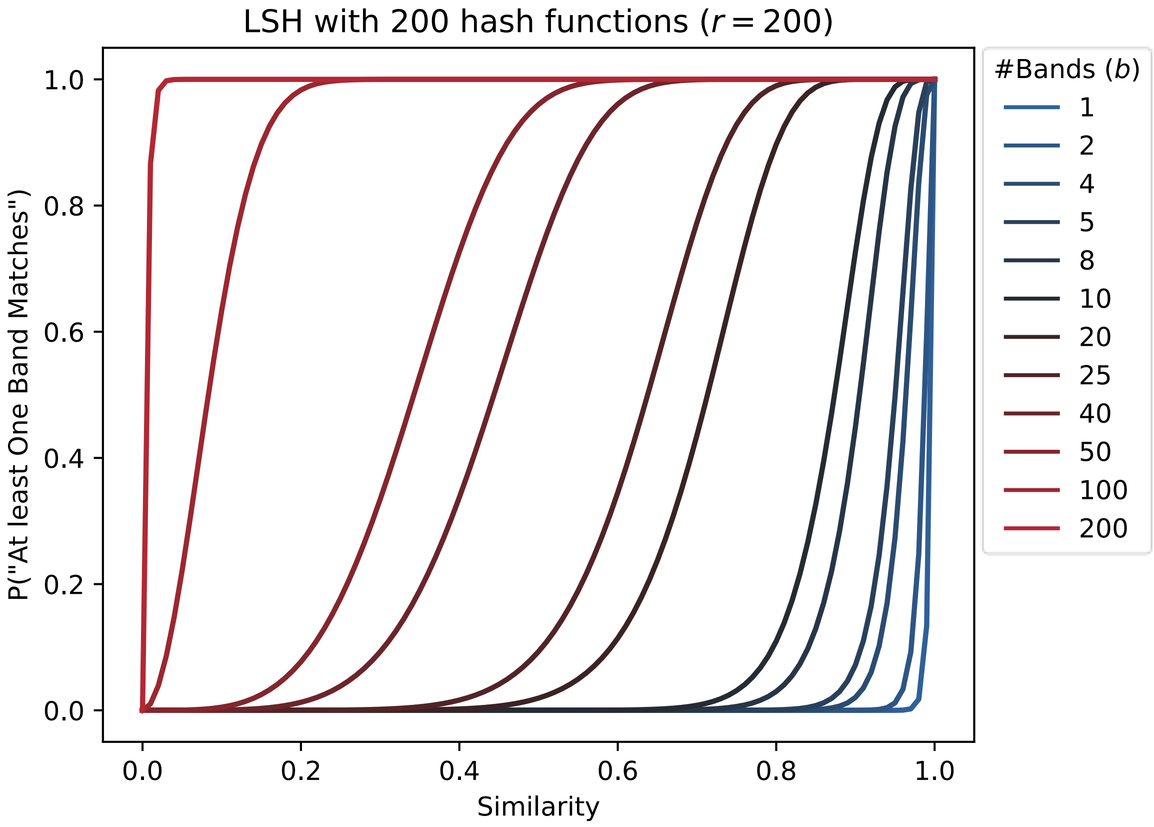

Supplementary Figure 6: *Probability of two signatures matching in at least one band of LSH with 200 hash functions.*

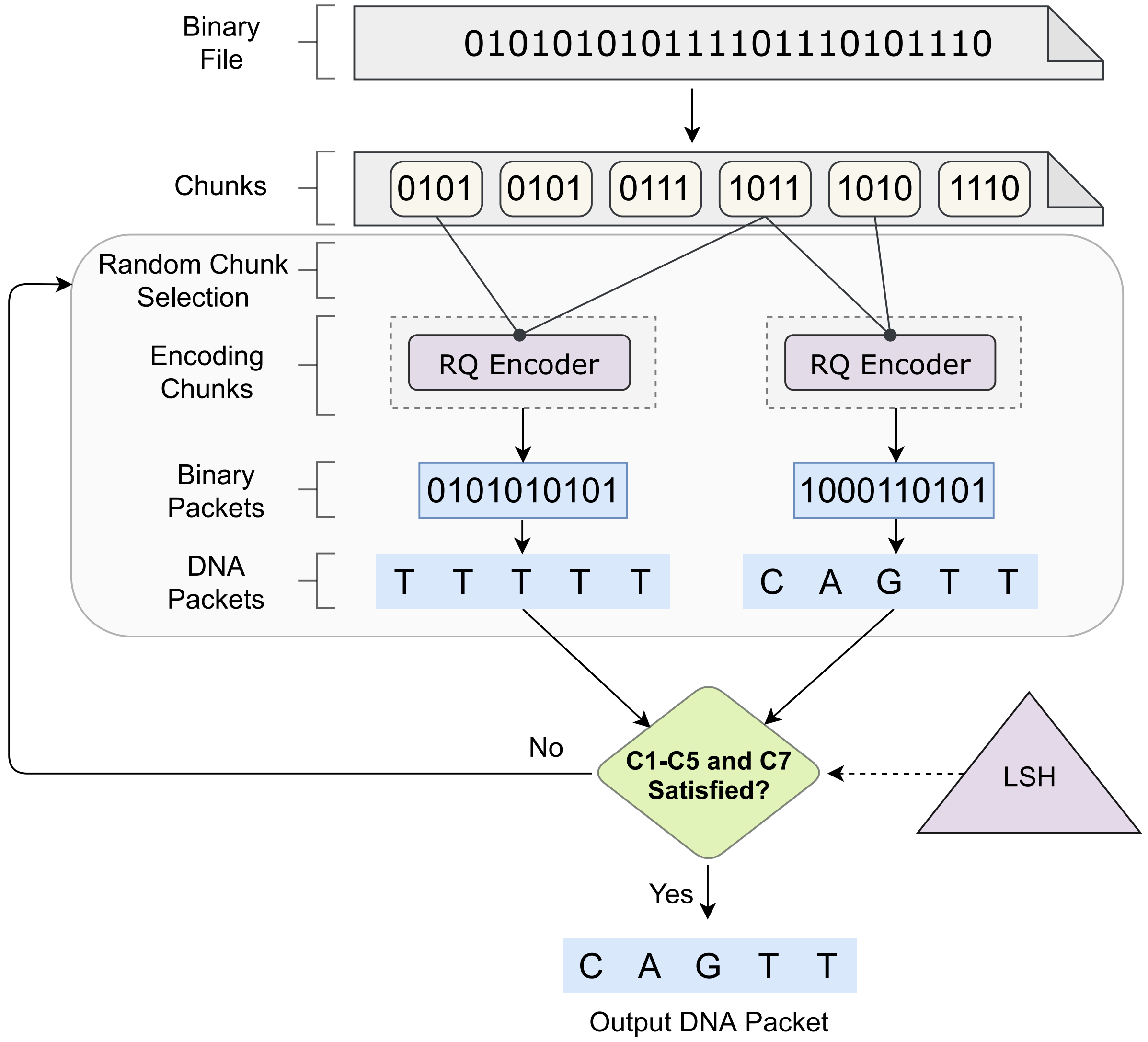

Supplementary Figure 7: *Utilizing RaptorQ code and to compute DNA packets that satisfy given constraints efficiently.*

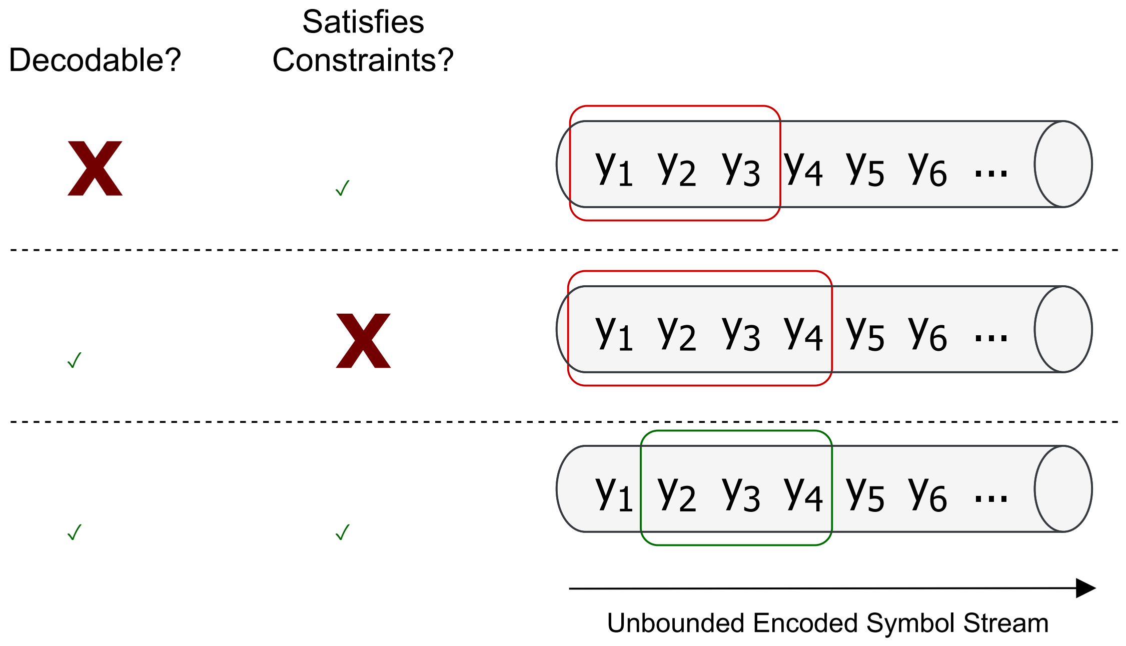

Supplementary Figure 8: *Selection of encoded symbols that yield a more stable DNA strand and allow a perfect recovery.*

References

1. De Vito, S., Massera, E., Piga, M., Martinotto, L. & Di Francia, G. On field calibration of an electronic nose for benzene estimation in an urban pollution monitoring scenario. *Sensors and Actuators B: Chemical* **129**, 750-757 (2008).
